# Editorial: Thyroid and parathyroid surgery: new and emerging concepts

**DOI:** 10.3389/fsurg.2025.1645516

**Published:** 2025-10-06

**Authors:** Rita Laforgia, Giovanna Pavone, Ferdinando Massimiliano Anelli, Giovanni Docimo, Antonio Ambrosi, Angela Pezzolla, Nicola Tartaglia

**Affiliations:** 1 Azienda Ospedaliero Universitaria Consorziale Policlinico di Bari, Bari, Italy; 2 Universita Degli Studi di Foggia Dipartimento di Scienze Mediche e Chirurgiche, Foggia, Italy; 3 Universita Degli Studi Della Campania Luigi Vanvitelli, Caserta, Italy

**Keywords:** thyroid cancer, parathyroid carcinoma, hyperparathyoidism, minimally invasive thyroid surgery, minimally invasive parathyroid surgery

The diagnosis of and surgical approach to thyroid and parathyroid diseases have completely changed in recent decades due to the development of new technologies and increase in knowledge.

The number of detected thyroid nodules has increased significantly thanks to the ultrasound accuracy. In addition, the management of hyperparathyroidism has similarly evolved with an increase in endocrine diseases. Prompt recognition and treatment of thyroid and parathyroid disorders could have a beneficial impact on health outcomes and could reduce the economic and social burden associated with the treatment of advanced disease.

Treatment and management of thyroid disease has seen many advances over the past decade, with most developments related to technologies focusing on the reduction of complications. Devices developed for surgical dissection with improved hemostasis and reduced heat transfer (and hence collateral tissue damage) is of particular interest in thyroid surgery. Laryngeal nerve injury and parathyroid gland dysfunction are complications of surgery with potentially devastating consequences and hence have been an area of focus for recent technological developments.

Conventional thyroidectomy and parathyroidectomy are still considered the standard of care, but other innovative and minimally invasive techniques have been pioneered such as the gasless endoscopic transaxillary approach, bilateral axillary-breast approach (BABA), behind-the-ear (RA) facelift approach, transoral endoscopic thyroidectomy via vestibular approach (TOETVA), Minimally Invasive Video-Assisted Thyroidectomy (MIVAT), or parathyroidectomy (MIVAP). The role of multidisciplinary teams with an endocrine surgeon, endocrinologist, oncologist, nuclear radiologist, and radiotherapist is essential to correctly manage these patients.

The aim of this special issue was to focus on new and emerging concepts about Thyroid and Parathyroid Surgery, including surgical techniques with possible complications, referral center experience, oncological outcomes, and multidisciplinary approaches. The contributions from leading experts in this field have reached this objective, especially with state of the art reviews on minimally invasive surgery or novel approaches to thyroid and parathyroid cancer.

This topic about Endocrine Surgery achieved excellent results, with 11 manuscripts accepted of high quality and scientific level. The authors belong to referral centers for thyroid and parathyroid disease from different countries.

All the accepted manuscripts contribute to the improvement of literature in this field.

Pacilli et al. reported the importance of the surgical approach in cases of lymph node disease for recurrence differentiated thyroid carcinoma (DTC) and demonstrated the safety and effectiveness of the ultrasound-guided approach. The aim of this method is to identify all pathological nodes and to facilitate exact localization; the best outcome is to perform a curative surgery.

Sang et al. considered an innovative approach in parathyroidectomy by using artificial intelligence (AI) to recognize the parathyroid glands (PGs). This technique can be very useful for young inexperienced surgeons. The authors provided a retrospective study based on a new AI model to detect PGs, which demonstrated improved performance with superior accuracy and precision over other models.

Cianci et al. underlined the safety of the fine-needle aspiration cytology (FNAC) technique, performed with 25 G caliber needles. In fact, FNAC plays a crucial role in the diagnosis of thyroid nodules and is an easy and cost-effective method. The proposed technique by the authors is less painful for patients, with low complication rates.

Staibano et al. provided a systematic review and network meta-analysis of diagnostic test accuracy to identify the intraoperative parathyroid hormone (iPTH) criteria and post-gland excision timepoint. The main aim was to predict surgical cures for hyperparathyroidism and unify the existing iPTH monitoring criteria to address the lack of standardization in the timing of post-parathyroid gland excision.

He et al. conducted a comprehensive systematic review and meta-analysis on the application of carbon nanoparticles in thyroid surgery in order to detect intraoperative and/or metastatic lymph nodes and improve surgical skills while protecting the integrity and function of the parathyroid glands. The authors demonstrated that the application of carbon nanoparticles can effectively improve the effects of surgical treatment, reducing post-operative complications such as hypocalcemia and minimizing the need for repeat surgery for undetected intraoperative and metastatic nodes.

Ünlü et al. reported a non-randomized prospective clinical study on the comparison between the severity of surgical trauma in TOETVA vs. Conventional open thyroidectomy (COT), using the inflammatory surgical stress markers interleukine-6 (IL-6), C- reactive protein (CRP), and white blood cells (WBC). The secondary objective was the evaluation of operative time, post-operative pain, and hospital stay. The results suggested that the higher postoperative CRP level and VAS score were in in the TOETVA group balanced by a postoperative aesthetic satisfaction.

Jin et al. documented a very interesting clinical case on a non-recurrent laryngeal nerve (NRLN), well-known as a rare variation of the recurrent laryngeal nerve, with an overall incidence of 0.7%. This variation can lead to intraoperative nerve injury. The authors detected the NRLN during an areola approach for radical thyroidectomy after an accurate intraoperative search of the nerve.

Wang et al. reported a systematic review and meta-analysis on the global prevalence of secondary hyperparathyroidism (SHPT) due to chronic kidney disease (CKD). This review demonstrated a high prevalence of SHPT in patients with CKD, highlighting the importance of the therapeutic approach for primary care.

Yamashita et al. reported a prospective cohort study about the treatment of hypocalcemia after total thyroidectomy. This condition represents a comprehensive post-operative complication. The use of 1,25-dihydroxyvitamin D can act as a sudden therapy option for burden hypocalcemia.

Zhao et al. considered, with an accurate Mendelian randomization, the genetic connection between immune cell traits and Hashimoto's Thyroiditis. Their findings provide guidance and direction for future treatment and clinical research.

Laforgia et al. described a retrospective observational study about parathyroid carcinoma (PC), which represents less than 1% of malignant neoplasms among endocrinological diseases. PC is still considered a great challenge in terms of preoperative diagnosis, management, and treatment. A surgical approach represents the best option for PC in referral endocrine surgery units.

